# Chemotherapy and anti-HER2 therapy in metastatic breast cancer in pregnancy followed by surgical treatment

**DOI:** 10.3332/ecancer.2019.930

**Published:** 2019-05-14

**Authors:** Julia Berwart, Fedro A Peccatori

**Affiliations:** 1 Department of Gynaecology, Clemente Alvarez Emergency Hospital, Rosario, S2002QEA Argentina; 2 Fertility and Procreation Unit, European Institute of Oncology, IRCCS, 20141 Milan, Italy

**Keywords:** breast cancer, pregnancy, stage IV, surgical treatment, primary tumour

## Abstract

Between 5% and 10% of women have distant metastases when they receive a breast cancer (BC) diagnosis. Metastatic BC is associated with poor prognosis but advances in systemic treatments have improved survival rates in recent decades.

Debates about local primary tumour management in metastatic stages continue, but multiple studies have shown that primary tumour surgery can be beneficial.

BC is one of the most commonly diagnosed neoplasias during pregnancy. Treatment of pregnant BC patients should follow the standard treatment of young, non-pregnant patients as closely as possible.

We present the case of a young, pregnant patient with metastatic BC with a complete clinical response to chemotherapy followed by surgical treatment.

## Introduction

Breast cancer (BC) is the most commonly diagnosed cancer in women of childbearing age [1].

Between 5% and 10% of women have distant metastases when they receive a BC diagnosis [2].

Diagnosis of BC in pregnancy (BCP) is uncommon, representing 1%–5% of all diagnoses in young women, with an incidence of 1:3,000 pregnancies [3].

BCP normally appears at later stages than BC in non-pregnant patients, often due to delayed diagnosis [4].

We present the case of a young, pregnant patient with metastatic BC with a complete clinical response to chemotherapy followed by surgical treatment. We then present a bibliographic review.

## Case report

**Figure 1.**
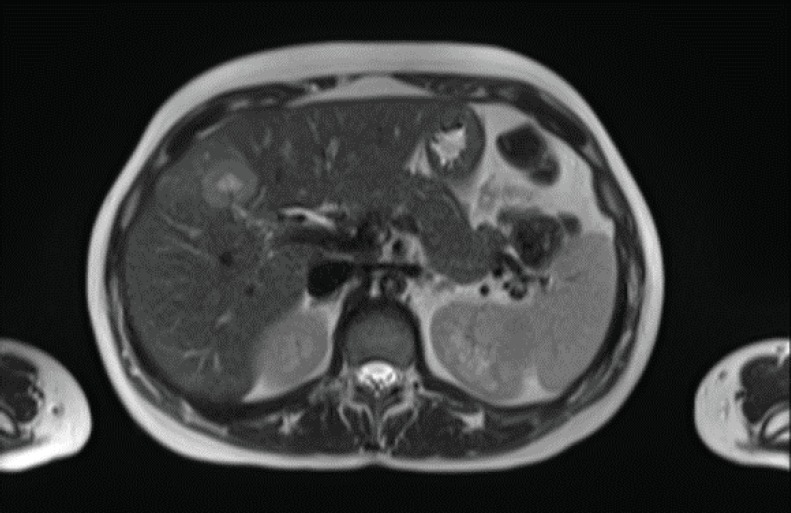
Whole-body nuclear magnetic resonance without contrast: images consistent with hepatic metastasis of segment V measuring 29 mm.

In 2018, a 33-year-old patient, Gravida 3, Caesarean 2, was evaluated in the 16th week of pregnancy. During the physical examination, tumour was palpated in the retroareolar region. This tumour was of increased consistency, with a maximum diameter of approximately 10 cm, irregular margins and clinically negative axilla. Needle biopsy: infiltrating ductal carcinoma, histological grade 3. Immunohistochemistry results: oestrogen receptor-positive (35%), progesterone receptor-positive (85%), HER2/neu-positive (Score 3+), Ki67 = 37%. Whole-body nuclear magnetic resonance without contrast found images consistent with hepatic metastasis of segment V measuring 29 mm (Figure 1) and millimetric metastases in the right iliac bone. Genetic test was negative (BRCA 1 and 2 not mutated).

The patient received chemotherapy after cardiological assessment with electrocardiogram (epirubicin 90 mg/m^2^ + cyclophosphamide 600 mg/m^2^) for four cycles during pregnancy. Treatment led to partial breast tumour remission and complete response of the lesion in the iliac region but the hepatic lesion increased in size. Dosages were calculated based on body surface area using patient weight at chemotherapy. The last cycle was administered 5 weeks before delivery to avoid maternal and child toxicity at birth.

This tumour was HER2-positive but anti-HER2 therapy is contraindicated during pregnancy. This case was thus discussed in a meeting of a multidisciplinary team, which decided to anticipate delivery. At 35 weeks and 4 days, 4 weeks after the last chemotherapy cycle, a healthy child was born weighing 2,345 g and measuring 49 cm. The patient had a scheduled caesarean section due to her obstetric history of two prior caesarean births. Pathological examination of the placenta was negative.

**Figure 2.**
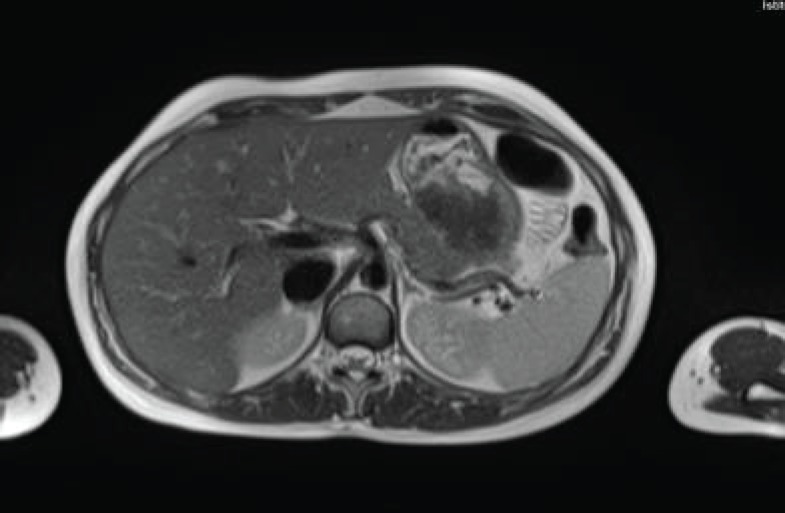
Whole-body nuclear magnetic resonance without contrast: complete remission of the hepatic lesion.

The patient continued treatment with trastuzumab and docetaxel for eight cycles. Whole-body nuclear magnetic resonance without contrast after chemotherapy ended found: breast lesion reduced by more than 50% and the size and functionality of the hepatic lesion reduced. The patient continued treatment with trastuzumab + pertuzumab, Decapeptyl and exemestane. Whole-body nuclear magnetic resonance 1 year later found: complete remission of hepatic (Figure 2) and bone lesions. Breast ultrasound found two millimetric formations measuring 10 and 7 mm in the upper outer quadrant of the right breast.

The multidisciplinary team and the patient met to discuss surgery. The patient then had a mastectomy with nipple-areola complex preservation, placement of a definitive breast prosthesis and contralateral mastopexy. A biopsy of sentinel lymph nodes was negative. Pathological examination found lobular *in situ* neoplasia (LIN2) associated with fibrosis.

The patient enjoys good health 22 months after diagnosis, is in excellent condition and free of disease, with a normal echocardiogram.

## Discussion

Metastatic BC has historically been considered incurable [5]. Primary therapy is systemic (chemotherapy, hormonal and biological treatment) [6, 7] and aims to reduce disease progression and improve quality of life.

Metastatic BC is associated with a poor prognosis but advances in systemic treatments have improved survival rates in recent decades [8–11].

BC is one of the most commonly diagnosed neoplasias during pregnancy [12]. Termination is generally not necessary and pregnancy does not worsen BC prognosis. However, patients with an unfavourable prognosis and advanced-stage disease in the first trimester should consider termination of the pregnancy [13].

Staging during pregnancy must consider the risks of ionising radiation, and bone scintigraphy and computed axial tomography are thus contraindicated. It is possible to use whole-body nuclear magnetic resonance without contrast in pregnant patients with locally advanced tumours [14].

Treatment of BCP should follow the standard treatment of young, non-pregnant patients as closely as possible.

Most treatments can be used safely, depending on the gestational age.

Chemotherapy is contraindicated during the first trimester of pregnancy due to its association with foetal malformations. It is recommended that, whenever possible, the second and third trimesters treatment follow guidelines for treating young, non-pregnant patients [15]. Various drugs (doxorubicin, epirubicin, docetaxel and paclitaxel) have shown a constant decrease in the plasma concentration curve and increased clearance during pregnancy. These changes can affect levels of free drug in the blood, which can reduce therapeutic effectiveness. However, no study has shown reduced therapeutic effectiveness or lower survival compared to non-pregnant patients [16]. Doses should not be modified during pregnancy and should be calculated based on actual patient weight. Pregnant patients should receive the last chemotherapy dose at least 4 weeks before birth to avoid administering high drug concentrations to infants at birth [17].

The same adjuvant or neoadjuvant treatment that non-pregnant patients receive, with anthracyclines and taxanes, is recommended after the first trimester. The recommended regimen is anthracyclines followed by taxanes. Epirubicin has better placental passage than doxorubicin and is, therefore, the treatment of choice [13]. The use of weekly paclitaxel is also preferable to docetaxel due to the lower haematological toxicity of the former [18].

Some agents, such as anti-HER2 drugs and endocrine therapy are contraindicated as they can cause foetal toxicity. Trastuzumab is associated with oligo-anhydramnios when administered for long periods in the second and third trimesters [19].

One of the most crucial factors that determine foetal health is gestational age at delivery. Trastuzumab should, therefore, be avoided before 37 weeks, but each case requires a personalised discussion. Our patient had a lack of hepatic response and could receive anti-HER2 treatment after pregnancy. Based on those factors, her pregnancy was ended at 35 weeks, after full foetal pulmonary development.

Breastfeeding is generally not recommended during chemotherapy as drugs are excreted in human milk. Excretion depends on the capacity of the drug to bind to plasma proteins, as well as its ionisation and liposolubility [20].

Breastfeeding becomes possible 3 weeks after chemotherapy ends. One study compared women who underwent chemotherapy during pregnancy with non-exposed women. That study found a significant difference in patient perception of reduced milk supply and a greater need to supplement the diets of their infants [21].

Surgery in stage IV has historically been limited to palliative treatment of primary tumour complications, such as bleeding, ulceration and infections at the tumour site (surgery described as a ‘toilet’ mastectomy); or for complications at other sites (spinal cord compression, pathological bone fractures, resection of single bone or brain metastases and pleurodesis). Debates about the impact of stage IV primary tumour surgery on survival continue.

It has been shown that controlling other types of primary tumours, such as metastatic renal, colorectal and gastric cancers and melanomas, can improve survival [22–25]. Removing the primary tumour may have an immunomodulating effect, reducing tumour load, eliminating a possible source of metastasis or reducing the likelihood that potentially resistant cells will develop [26, 27].

It has also been suggested that surgically resecting the primary tumour can stimulate the growth of metastases and shorten survival [28, 29]. Other hypotheses assume that BC spreads via blood and the lymphatic vessels. Local control is thus not important as it does not affect the development of subsequent metastases [30].

Multiple retrospective studies suggest that primary tumour surgery in patients with stage IV BC can be beneficial [31–34].

One meta-analysis evaluated ten retrospective studies with a total of 28,693 patients. That study suggested that survival times improved in selected patients who underwent primary tumour surgery (40%) compared to patients who only received systemic therapy (22%) [35]. This improvement in survival may reflect selection bias: younger, healthier patients with low tumour loads, oligometastasis or metastasis to a more favourable site and tumours with better biological profiles [36].

There are six randomised and controlled studies underway in various countries: the United States, Austria, the Netherlands, Japan, India and Turkey. We now have results from two of these studies.

The first is an Indian study that selected 350 patients with stage IV BC who responded to initial systemic therapy. These patients were randomised into two groups, one that received local-regional treatment (LRT) and another group that did not. This study found no differences in overall 2-year survival between the two groups: 43% in the non-LRT group and 42.9% in the LRT group [37].

The second study was conducted in Turkey and randomised 278 patients: one group received only systemic therapy and the other received LRT followed by systemic therapy. This study found a higher 5-year overall survival rate for the second group, 41.6%, versus 24% for the first group. This study also found that the patients who survived longer were those under age 55 with positive hormone receptors, HER2-negative disease and single bone metastases [38].

## Conclusion

Metastatic BC can be treated effectively during pregnancy. Anthracycline and taxane chemotherapy administered during pregnancy is effective for patients and safe for foetuses. Conversely, trastuzumab, pertuzumab and endocrine therapy are contraindicated as they can cause foetal harm.

LRT merits strong consideration in patients like ours, who have the good general condition, systemic oligometastatic disease and favourable tumour biology. However, clinicians must also consider the age of the patient. A multidisciplinary team which includes oncologists, breast specialists, obstetricians, paediatricians, surgeons, psychologists and nurses trained to provide comprehensive care is vital.
